# *Tc-MYBPA* an Arabidopsis TT2-like transcription factor and functions in the regulation of proanthocyanidin synthesis in *Theobroma cacao*

**DOI:** 10.1186/s12870-015-0529-y

**Published:** 2015-06-25

**Authors:** Yi Liu, Zi Shi, Siela N. Maximova, Mark J. Payne, Mark J. Guiltinan

**Affiliations:** Huck Institute of Life Sciences, The Pennsylvania State University, University Park, PA 16802 USA; Department of Horticulture, The Pennsylvania State University, 422 Life Sciences Building, University Park, PA 16802 USA; Hershey Center for Health and Nutrition, The Hershey Company, 1025 Reese Ave., Hershey, PA 17033 USA; Present address: Cellular & Molecular Pharmacology, University of California, San Francisco, Mission Bay Campus, Genentech Hall, N576/Box 2280, 600 16th Street, San Francisco, CA 94158 USA

**Keywords:** *Theobroma cacao*, Proanthocyanidin, Transcription factor, TT2

## Abstract

**Background:**

The flavan-3-ols catechin and epicatechin, and their polymerized oligomers, the proanthocyanidins (PAs, also called condensed tannins), accumulate to levels of up to 15 % of the total weight of dry seeds of *Theobroma cacao* L. These compounds have been associated with several health benefits in humans. They also play important roles in pest and disease defense throughout the plant. In Arabidopsis, the R2R3 type MYB transcription factor TT2 regulates the major genes leading to the synthesis of PA.

**Results:**

To explore the transcriptional regulation of the PA synthesis pathway in cacao, we isolated and characterized an R2R3 type MYB transcription factor MYBPA from cacao. We examined the spatial and temporal gene expression patterns of the *Tc-MYBPA* gene and found it to be developmentally expressed in a manner consistent with its involvement in PAs and anthocyanin synthesis. Functional complementation of an Arabidopsis *tt2* mutant with *Tc-MYBPA* suggested that it can functionally substitute the Arabidopsis *TT2* gene. Interestingly, in addition to PA accumulation in seeds of the *Tc-MYBPA* expressing plants, we also observed an obvious increase of anthocyanidin accumulation in hypocotyls. We observed that overexpression of the *Tc-MYBPA* gene resulted in increased expression of several key genes encoding the major structural enzymes of the PA and anthocyanidin pathway, including *DFR* (dihydroflavanol reductase), *LDOX* (leucoanthocyanidin dioxygenase) and *BAN* (*ANR*, anthocyanidin reductase).

**Conclusion:**

We conclude that the *Tc-MYBPA* gene that encodes an R2R3 type MYB transcription factor is an Arabidopsis *TT2* like transcription factor, and may be involved in the regulation of both anthocyanin and PA synthesis in cacao. This research may provide molecular tools for breeding of cacao varieties with improved disease resistance and enhanced flavonoid profiles for nutritional and pharmaceutical applications.

**Electronic supplementary material:**

The online version of this article (doi:10.1186/s12870-015-0529-y) contains supplementary material, which is available to authorized users.

## Background

**Fig. 1:**
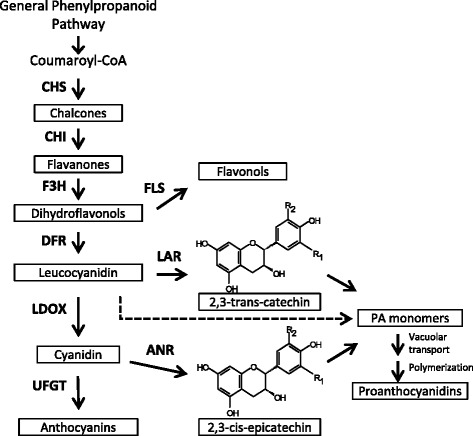
Working model of Anthocyanin and Proanthocyanidin synthesis pathway adapted from [23]. Enzymes are represented in uppercase bold letters; the products in the pathway are given in black boxes. The enzymes involved in the pathway are shown as follows: CHS, chalcone synthase; CHI, chalcone isomerase; F3H, flavanone-3β-hydroxylase; DFR, dihydroflavonol-4-reductase; LDOX, leucoanthocyanidin dioxygenase; FLS, flavonol synthase; LAR, leucoanthocyanidin reductase; ANR, anthocyanidin reductase; and UFGT, UDP-Glc:flavonoid-3-O-glucosyltransferase

Proanthocyanidins (PAs) are a subgroup of a large class of plant secondary metabolites known as flavonoids. Due to their important roles in plant defense and their beneficial role in human health, our understanding of PAs as well as the general flavonoid biosynthetic pathway has greatly improved in the past decades [1–5]. A general PA synthesis pathway is summarized in Fig. 1. The mechanisms regulating the transcription of the flavonoid biosynthetic pathway genes are well studied in the model systems Arabidopsis (*Arabidopsis thaliana*) and maize *(Zea mays*) [6]. Transcriptional regulation of the genes encoding the key enzymes of the flavonoid pathway is mediated by members of three protein families: the R2R3-MYB transcription factors, the MYC-like basic helix-loop-helix (bHLH) proteins and the WD40 repeat proteins [6–8].

The regulation of proanthocyanidin (PA) synthesis has been well characterized by the analysis of transparent testa (*tt*) mutants that fail to accumulate PAs in the seed coat [6, 9]. Three TT loci, *TT2*, *TT8* and *TTG1*, which encode R2R3-MYB, bHLH and WD40 repeat proteins respectively, are necessary for proper temporal and spatial accumulation of PAs [6]. The combinatorial interactions of different members from these three protein families determine the specificity of target gene activation [4, 6, 10, 11]. This interaction has been shown for several flavonoids synthesis regulators isolated from Arabidopsis [4, 6, 10, 11], *Zea mays* [12, 13] and *Petunia hybrida* [14–16]. The three proteins interact and form a ternary transcriptional protein complex to activate “late” PA-specific genes including *DFR* (dihydroflavanol reductase), *LDOX* (leucoanthocyanidin dioxygenase, also called ANS, anthocyanin synthase) and *BAN* (*ANR*, anthocyanidin reductase*)* [10, 11, 17, 18]. Another three TT loci, *TT16*, *TT1* and *TTG2* that encode a MADS box protein, a zinc-finger protein and a WRKY transcription factor, respectively, are also important for PA synthesis [6]. These proteins have been shown to regulate the expression of BAN protein through a posttranscriptional mechanism and thus are involved in the differentiation of PA-accumulating cells [6].

The *TT2* gene product (TT2) is a key regulator of PA synthesis and confers target gene specificity to the MYB-bHLH-WD40 complex. It is specifically expressed in PA-accumulating cells in Arabidopsis but can induce ectopic expression of the *BAN* gene when constitutively expressed in the presence of a functional TT8 protein [10]. TT2 belongs to the large R2R3-MYB protein family that has 133 members in Arabidopsis. These proteins are typically involved in many aspects of plant secondary metabolism, plant cell identity and cell fate determination [19, 20]. Members of the R2R3-MYB protein family are characterized by the presence of two highly conserved head-to-tail MYB motifs in the N-terminal region, the R2 and R3 repeats, although their C-terminal regions are very divergent. Each of the R2R3 repeats consists of three α-helices [12]; helix 3 of each motif is involved in interaction with DNA and helix 1 of the R3 repeat is important for corresponding bHLH recognition.

In addition to Arabidopsis, the TT2-like PA-specific R2R3-MYB transcription factors (TFs) have been characterized in grape (*Vitis vinifera*), *Lotus* (*Lotus japonicus*), poplar (*Populus tremuloides*), persimmon (*Diospyros kaki*), clover (*Trifolium arvense*) and *Medicago* (*Medicago truncatula*) [21–27]. In grape, two TT2-like MYB TFs (VvMYBPA1 and VvMYBPA2) have been identified {Bogs, 2007 #594}{Terrier, 2009 #9}. These TFs exhibit tissue-specific functions in inducing PA structural gene expression and synthesis: VvMYBPA1 is mainly expressed in seeds; and VvMYBPA2 is mainly in expressed in exocarp of young berries and in the leaves. Similar observations were reported in *Lotus*, in which three copies of TT2-like R2R3-MYB TFs were identified that differed in organ-specific expression and responsiveness to stress {Yoshida, 2008 #775}. Each of the TFs mentioned above is capable of activating the *ANR* promoter in transient reporter assays. In poplar, a *MYB134* gene encoding a TT2-like TF was recently shown to be responsive to wounding, pathogen presence and UV-B irradiation, consistent with the biological roles of PAs in anti-herbivore, anti-pathogen and UV damage protection {Mellway, 2009 #823}. Overexpression of MYB134 in poplar resulted in transcriptional activation of the genes encoding enzymes of the full PA biosynthesis pathway from PAL1 to ANR and LAR, but not FLS, which is specific to flavonol synthesis.

There are a variety of plant-based foods and beverages that serve as natural sources of flavonoids, including cacao, red wine, grape, apple and cranberries. Among those, cacao has an extraordinarily high amount of flavonoid, especially PAs [28], which make up about 10–14 % of dry weight in mature beans [29]. The development of cacao and flavonoid (mainly anthocyanins) synthesis has been described previously [30]. The development of cacao fruits can be divided into three phases [31]. Following pollination and fertilization, the first phase of fruit development is initiated and fruit begins to expand slowly at a rate of about 30–40 cm^3^/ week [32]. This phase lasts 6–7 weeks until the first division of the fertilized egg, which initiates the second phase of pod development. At the second phase, fruits expand more rapidly at a rate of about 110–130 cm^3^/week, and embryos enlarge but remain unpigmented till they reach the length of ovules at about 14–16 weeks after pollination [31, 33]. When the fruits are 14–16 weeks old, the pericarp begins to change color from green to orange (in Scavina 6), denoting onset of the third phase, ripening. Ripe pod color varies from bright red, purple, green, yellow and multi-colored patterns, dependent on genotype. During the third phase, the increase in the fruit external dimensions gradually slows and finally ceases. The seeds begin to solidify and their dry weight increases rapidly at a rate of about 20–40 mg/day. Seed length remains constant as they continue to accumulate anthocyanins and gradually darken until maturity at about 20 weeks after pollination [30–33].

This research describes the isolation and characterization of a cacao gene, *Tc-MYBPA,* which encodes an R2R3-MYB transcription factor involved in regulating the biosynthesis of cacao PAs. Constitutive expression of *Tc-MYBPA* in the Arabidopsis *tt2* mutant not only successfully complemented its primary phenotype (a PA-deficient seed coat) but also resulted in increased anthocyanin accumulation in young seedlings, suggesting that *Tc-MYBPA* may regulate both the anthocyanin and PA pathways in cacao.

## Results

### The Cacao *Tc-MYBPA* gene encodes an R2R3-MYB transcription factor

Four putative *Tc-MYBPA* cDNA sequences were identified in a collection of *Theobroma cacao* expressed sequence tags (ESTs) [34] by querying the cacao ESTtik database (http://esttik.cirad.fr/) with the protein sequence of Arabidopsis TT2 (accession no. Q9FJA2). This cacao EST database contains 56 cDNA libraries constructed from different organs; two main genotypes and different stress conditions thus could be considered as an exhaustive collection of cacao expressed genes [34]. ESTs showing sequence similarity to the *TT2* gene were assembled into a contig to recover full-length open reading frames (ORFs) by alignment with cDNAs of homologous genes from other species and predictions from the ORF Finder program (www.ncbi.nlm.nih.gov/projects/gorf/). The full-length coding sequence of *Tc-MYBPA* was amplified by RT-PCR using cDNAs isolated from young leaves of cacao (Scavina 6), in which PAs are actively synthesized and accumulated [35]. The isolated ORF was named *Tc-MYBPA* (accession no. GU324346). By searching the newly assembled cacao genome [36], we identified the *Tc-MYBPA* gene (Tc01_g034240) that is 1477-bp long with two exons. It is not associated with any currently identified quantitative trait loci (QTL) related to flavonoids. However, the *Tc-MYBPA* is very closely associated with 7 out of 17 DFR orthologous genes located near the bottom of chromosome 1. We also searched the whole cacao genome with the protein sequence of Arabidopsis TT2 to check if there are other possible homologues genes. The search revealed 7 candidate genes with higher score than *Tc-MYBPA* (Additional file 1: Figure S1). However, we didn’t find any confident hits by searching their putative protein sequences back to the cacao EST database. Considering that this EST database contains a variety of tissues that have been shown to synthesize and accumulate PAs [34, 37], including leaves, roots, flowers, pods, seeds, and seed testa, the 7 candidate genes maybe be peudogenes and not express at all.

The 864-bp ORF of *Tc-MYBPA* encodes a protein of 287 amino acids that shares 68 % identity with grape *VvMYBPA1*. A protein sequence alignment of *Tc-MYBPA* with other PA- and anthocyanin-regulating MYB proteins revealed that *Tc-MYBPA* contains an N-terminal R2R3 repeat that corresponds to the DNA-binding domain of plant MYB-type proteins (Fig. 1a). Like the high sequence similarity observed between the R2R3 repeat regions shared by 126 members of Arabidopsis [19, 38], the *Tc-MYBPA* R2R3 repeat region is highly conserved when compared to other plant R2R3 MYBs. The *Tc-MYBPA* N-terminal region also contains the [D/E]LX_2_[R/K]X_3_LX_6_LX_3_R motif for interaction with bHLH partners in the R3 repeat region [12], whereas the C-terminal region shows little homology to the MYB proteins included in this comparison.

To investigate these relationships more closely, a phylogenetic tree was constructed using the full-length amino acid sequences of *Tc-MYBPA* and sequences of all functionally tested MYBs involved in regulating proanthocyanidin and anthocyanin biosynthesis, as well as MYBs associated with several other biological processes (Fig. 1b). By searching the cacao EST database using tBLASTn with the protein sequence of putative cacao MYB *Tc-MYBPA* as the query, three EST contigs (CL8212Contig1, CL2621Contig1 and CL158Contig1) containing MYB-like proteins were also identified as the next best cacao matches to *Tc-MYBPA*. The results show that the putative cacao proanthocyanidin regulatory protein *Tc-MYBPA* is most closely related to the grape PA regulatory MYB protein VvMYBPA1 and clusters in the same clade with all the anthocyanidin and proanthocyanidin regulatory MYB proteins.

**Fig. 2:**
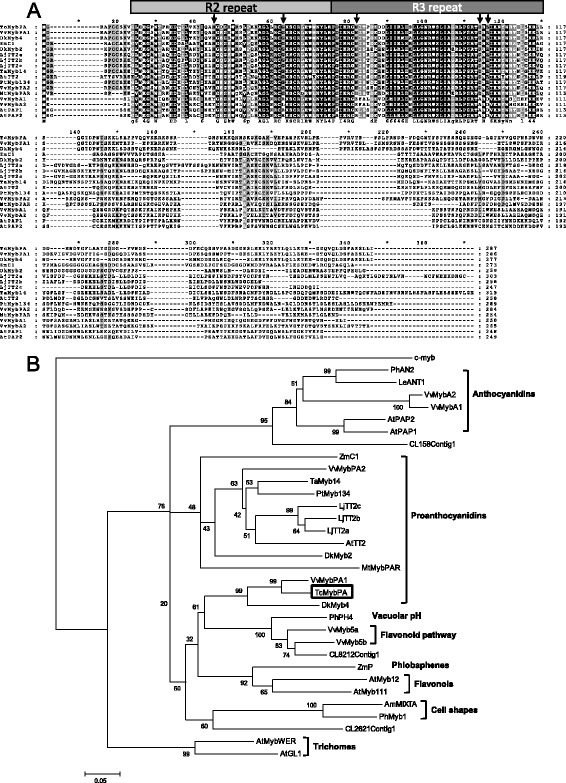
Comparison of the amino acid sequences of *Tc-MYBPA* and various plant MYB transcription factors. **a** Alignment of the deduced amino acid sequences of the R2R3-MYB proteins functioning in anthocyanin and PA synthesis, including *Tc-MYBPA* (cacao), ZmC1 (maize), VvMybPA1, VvMybPA2, VvMybA1, VvMybA2 (grape), PtMyb134 (poplar), LjTT2a, LjTT2b, LjTT2c (*Lotus*), and Arabidopsis regulators AtTT2, AtPAP1 and AtPAP2. The R2 and R3 repeats of the MYB domain are indicated above the alignment. Identical amino acids are indicated in black, similar amino acids in gray. Arrowheads indicate amino acids that are conserved in all PA-regulating MYBs but absent in anthocyanin-regulating MYBs. Sequences were aligned using the ClustalW program and were displayed using the GeneDoc program. **b** Phylogenetic tree showing selected plant MYB transcription factors from GenBank. Human c-myb is included as an outgroup. Functions of the MYB proteins are given on the right side in bold. The alignment was performed using the ClustalW program and the tree was constructed using the neighbor-joining algorithm of the MEGA package (Version 3.1). The scale bar represents 0.1 substitutions per site and the numbers next to each node are bootstrap values from 1000 replicates. The GenBank accession numbers of the MYB proteins are as follows: AtGL1 (P27900), ZmP (P27898), ZmC1 (AAA33482), VvMybA1 (BAD18977), VvMybA2 (BAD18978), AtPAP1 (AAG42001), PhAN2 (AAF66727), LeANT1 (AAQ55181), OsMyb4 (T02988), AmMixta (CAA55725), AtMyb111 (AAK97396), AtMyb12 (NM_130314), PmMybF1 (AAA82943), PhPH4 (AAY51377), AtPAP2 (AAG42002), AtMybWER (CAC01874), VvMyb5a (AAS68190), VvMYB5b (Q58QD0), VvMYBPA1 (AM259485), VvMybPA2 (ACK56131), c-myb (AAB49039), PtMyb134 (FJ573151), PhMyb1 (Z13996), LjTT2a (AB300033), LjTT2b (AB300034), LjTT2c (AB300035), AtTT2 (Q2FJA2), MtMybPAR (ADU78729), TaMyb14 (AFJ53053) Also included in the tree are one putative cacao PA specific MYB (*Tc-MYBPA*), and three MYB-like proteins found in the cacao EST collections (CL158Contig1, CL8212Contig1 and CL2621Contig1)

This clade also includes VvMYB5a and VvMYB5b from grape, which are involved in regulating the entire flavonoid pathway, and PhPH4 from petunia, which is involved in regulating vacuolar pH. R2R3-type MYB proteins that regulate other biochemical and physiological processes such as phlobaphene and flavonol synthesis, cell shape determination and trichome development clustered into separate subgroups. The other three cacao MYB-like proteins cluster together with MYBs that have functions other than proanthocyanidin regulation, such as flavonoid pathway regulation (CL8212Contig1), cell shape determination (CL2621Contig1) and anthocyanidin synthesis regulation (CL158Contig1). ZmC1, the maize anthocyanin synthesis regulator that was shown to activate the Arabidopsis *ANR* promoter [11], clustered together in the same subgroup with AtTT2 and VvMYPPA2, which are functionally verified PA regulators. This was consistent with the protein alignment analysis in which ZmC1 was more similar to PA regulatory MYBs than to anthocyanin regulatory MYBs. Protein alignment also revealed that some conserved amino acids present in the N-terminal region of *Tc-MYBPA* as well as all PA regulatory MYB proteins and ZmC1 were absent in all the other anthocyanin MYB factors (Fig. 2); this could indicate similarity in function. These included, according to position on *Tc-MYBPA*, His32, Gly50, Ile70, Asp101, Glu103, and Ile104.

In summary, the *Tc-MYBPA* protein sequence includes conserved R2R3 regions typical of plant MYB transcription factors. Moreover, in *Tc-MYBPA*, we were able to detect conserved amino acid homologies shared with all the TT2-like MYB regulators but absent in anthocyanin regulators. These conserved amino acids appear to be specific to this clade and may be used to identify candidate PA-specific MYB regulators from other plant species.

### Expression of *Tc-MYBPA* correlates with PA accumulation in *Theobroma cacao*

We have previous identified and functionally verified key PA biosynthesis structural genes *TcANR, TcANS* and *TcLAR* [37]. A scan of the promoter sequences in the PALACE database [39] of these PA synthesis genes revealed several target motifs of Myb transcription factors on each of them (Additional file 1: Figure S2). Interestingly, MYBCORE, the key cis-regulatory element for binding PA synthesis regulating Myb transcription factors [40], was found in all of them, suggesting that they could all be downstream targets of the putative *Tc-MYBPA*. To assess the involvement of *Tc-MYBPA* in PA biosynthesis, the expression of the putative *Tc-MYBPA* gene was examined in tissue samples from different developmental stages of leaves, flowers and pods in which PAs accumulate. In addition, the expression of the cacao PA biosynthesis structural genes *TcANR, TcANS* and *TcLAR* were also examined*.*

**Fig. 3:**
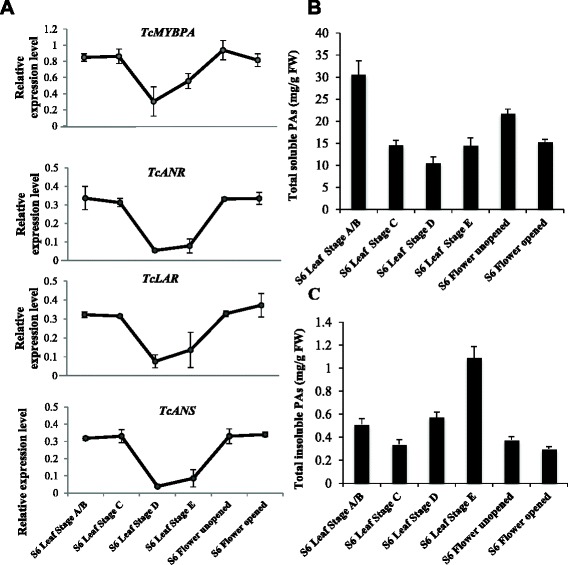
Expression of *Tc-MYBPA*, *TcANR*, *TcANS* and *TcLAR* genes and accumulation of PAs in *Theobroma cacao* (Scavina 6; S6) leaves and flowers at various developmental stages. **a** Transcript levels of *Tc-MYBPA*, *TcANR*, *TcANS* and *TcLAR*. Expression was determined by semi-quantitative RT-PCR and was calculated relative to the expression of *TcActin* in each sample. **b** Levels of soluble PAs expressed as mg PA per g of fresh weight. **c** Levels of insoluble PAs expressed as mg PA per g of fresh weight. All data are presented as means ± SE, for gene expression data, n ≥ 3, for PA level data, n ≥ 5. FW, Fresh weight

A strong positive correlation of expression levels of the putative *Tc-MYBPA* and the structural genes was observed in all tissues. The steady state levels of *Tc-MYBPA, TcANR*, *TcANS* and *TcLAR* transcripts were highest in young leaves and decreased in older leaves (Fig. 3a). Relatively high levels were present in flower tissues. We also measured the accumulation of total soluble PAs (including PA polymers as well as monomers) and insoluble PAs in the different tissues by DMACA assay and butanol-HCl assay respectively (described in details in Methods). Both cacao leaves and flowers contained significant levels of PAs. The highest total soluble PAs were detected in the youngest leaves (about 30 mg procyanidin B2 equivalent/g fresh weight (FW), Fig. 3b). Much lower amounts were detected in older leaves. Total insoluble PAs were relatively lower in young leaves and continued to increase as the leaves aged and became harder. Insoluble PAs reached their maximum level in lignified stage E leaves (about 1.2 mg cyanidin equivalent/g FW, Fig. 3c). PA levels were also considerable in flowers, with higher soluble PAs levels observed in unopened flowers than in opened flowers, and the levels of the insoluble fraction relatively the same in the two stages of flower development (Fig. 3b, c).

**Fig. 4:**
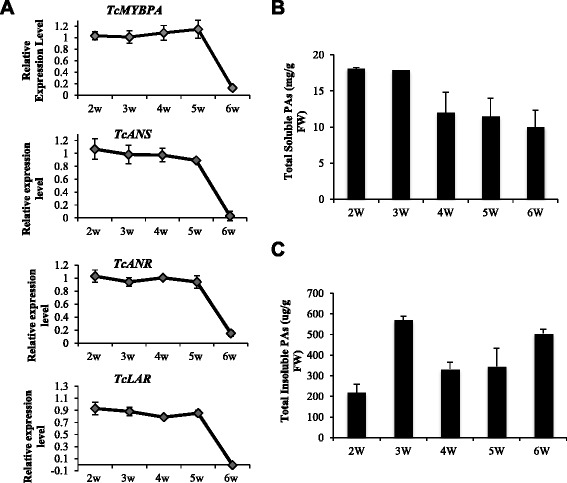
Expression of *Tc-MYBPA*, *TcANR, TcANS* and *TcLAR* genes and accumulation of PAs in whole pods of *Theobroma cacao* (Amelonado) during early stages of pod development (from 2 to 6 weeks after pollination). **a** Transcript levels of *TcANR*, *TcANS* and *TcLAR*. Expression was determined by semi-quantitative RT-PCR and was calculated relative to the expression of *TcActin* in each sample. **b** Levels of total soluble PAs expressed as mg PAs per g of fresh weight. **c** Levels of total insoluble PAs expressed as μg PAs per g of fresh weight. All data are presented as means ± SE. For gene expression data, n ≥ 3, for PA accumulation data, n ≥ 5. FW, Fresh weight

Figure 3 shows both the expression patterns of *Tc-MYBPA*, *TcANR*, *TcANS* and *TcLAR* (Fig. 3a) and PA levels in whole cacao pods early in their development when the pods are too small to separate ovules and exocarp (Fig. 4b, c). The expression of both *Tc-MYBPA* and the three PA structural genes shared a similar pattern. Their expression was relatively high at two weeks after pollination (WAP) and remained high at 5 WAP, followed by a significant decrease at 6 WAP (Fig. 4a). Levels of soluble PAs were already close to maximum (approximately 18 mg procyanidin B2 equivalent/g FW) at the earliest sampling time point (Fig. 4b), whereas insoluble PAs reached maximum levels at 3 WAP (Fig. 4c).

**Fig. 5:**
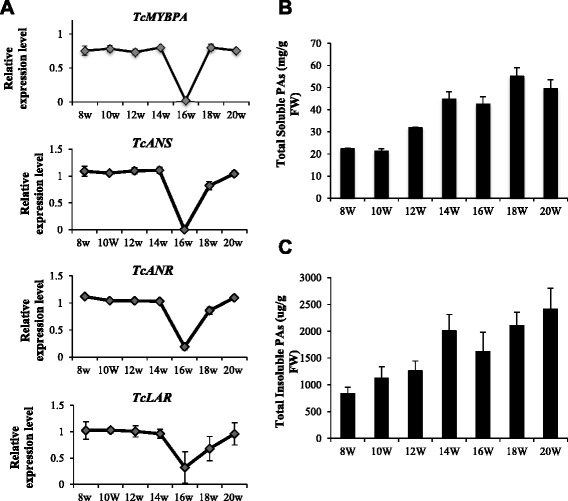
Expression of *Tc-MYBPA*, *TcANR, TcANS* and *TcLAR* genes and accumulation of PAs in pod exocarp of *Theobroma cacao* (Amelonado) during pod development (from 8 to 20 weeks after pollination). **a** Transcript levels of *Tc-MYBPA*, *TcANR*, *TcANS* and *TcLAR*. Expression was determined by semi-quantitative RT-PCR and was calculated relative to the expression of *TcActin* in each sample. **b** Levels of total soluble PAs expressed as mg PAs per g of fresh weight. **c** Levels of total insoluble PAs expressed as μg PAs per g of fresh weight. All data are presented as means ± SE, for gene expression data, n ≥ 3, for PA level data, n ≥ 5. FW, Fresh weight

At 8 WAP, the pods were large enough to allow dissection into exocarp and ovule samples for separate analysis. Expression patterns of *Tc-MYBPA*, *TcANR*, *TcANS* and *TcLAR* genes and PA levels in cacao pod exocarp tissues were examined at two-week intervals, from 8 WAP to 20 WAP, when pods fully ripened. The expression of all four genes examined was similar (Fig. 5a). They were all relatively high from 8 WAP to 14 WAP but decreased significantly at 16 WAP, increasing again at 18 WAP and reaching a maximum at 20 WAP. In accordance with gene expression patterns, the deposition of both soluble and insoluble PAs continued to increase during the development of the pods, reaching a maximum (soluble PA at approximately 50 mg procyanidin B2 equivalent/g FW; insoluble PA at approximately 2.5 mg cyanidin equivalent/g FW) around the time of ripening (Fig. 5b, c), while a pause of the PA accumulation occurred at 16 WAP, at which time point, soluble PAs were about the same level as 14 WAP and insoluble PAs slightly decreased.

**Fig. 6:**
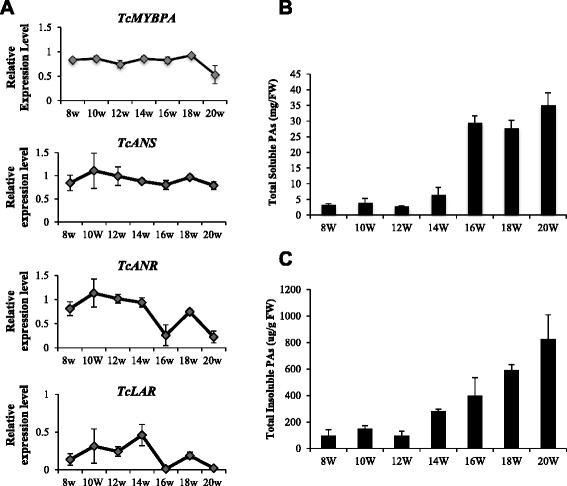
Expression of *Tc-MYBPA*, *TcANR, TcANS* and *TcLAR* genes and accumulation of PAs in ovules of *Theobroma cacao* (Amelonado) during pod development (from 8 to 20 weeks after pollination). **a** Transcript levels of *Tc-MYBPA*, *TcANR*, *TcANS* and *TcLAR*. Expression was determined by semi-quantitative RT-PCR and was calculated relative to the expression of *TcActin* in each sample. **b** Levels of total soluble PAs expressed as mg PAs per g of fresh weight. **c** Levels of total insoluble PAs expressed as ug PAs per g of fresh weight. All data are presented as means ± SE, for gene expression data, n ≥ 3, for PA level data, n ≥ 5. FW, Fresh weight

Unlike the co-regulated pattern of gene expression in exocarp, the expression pattern of *Tc-MYBPA* and *TcANS* differed quite significantly from that of *TcANR* and *TcLAR* in ovules (Fig. 6a). The expression of *TcANR* and *TcLAR* in ovules was quite similar, maintaining relatively high levels before 14 WAP but significantly decreasing at 16 WAP, then increasing at 18 WAP and dropping again at 20 WAP. The overall expression level of *TcLAR* was lower than that of *TcANR*. In contrast, neither *Tc-MYBPA* nor *TcANS* expression decreased at 16 WAP but remained relatively stable (0.7-1.2 relative to *TcActin*) throughout pod development, from 8 WAP to 20 WAP, although a slight increase did occur after 16 WAP followed by slight decrease at 20 WAP. The PA concentrations of both soluble and insoluble fractions in cacao ovules were lower than in exocarp (Fig. 6b, c). The ovule soluble PA accumulation was relatively low before 16 WAP and significantly increased at 16 WAP, reaching a maximum at 20 WAP (about 35 mg procyanidin B2 equivalent/g FW). However, throughout the development of ovules, insoluble PA levels increased at a relatively constant rate from 14 WAP.

The coordinated expression of *Tc-MYBPA* and *TcANS* suggest that *Tc-MYBPA* may contribute to the regulation of anthocyanin synthesis as well as PA synthesis. Nevertheless, the regulation of the PA-specific genes *TcANR* and *TcLAR* may also involve other transcription factors such as bHLH and WD40 repeat proteins whose interactions with *Tc-MYBPA* determine their specific expression patterns, which are slightly different from *TcANS.* To gain a better understanding of their regulation, further characterization and expression analysis of *bHLH* and *WD40* genes will be helpful.

### *Tc-MYBPA* complements the PA-deficient phenotype of the Arabidopsis *tt2* mutant

**Fig. 7:**
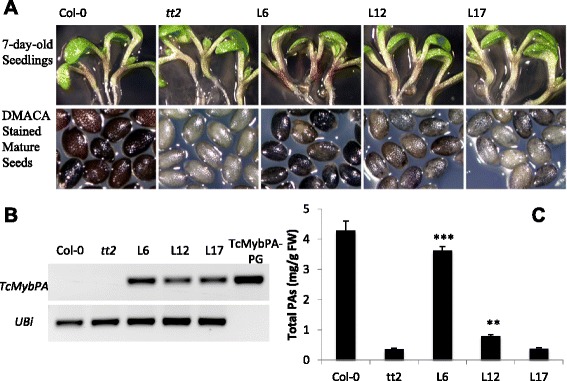
Complementation of the PA-deficient *tt2* mutant phenotype by constitutively expressing *Tc-MYBPA*. **a** 7-day old seedlings of and DMACA stained seeds from Col-0, the *tt2* mutant (SALK_005260) and three independent T2 transgenic lines of *tt- 35S:Tc-MYBPA*. The bar represents 1 mm. **b** RT-PCR analysis of *Tc-MYBPA* and *AtUbiquitin* transcripts in total RNA from the young seedlings shown in (**a**). PCR products from the *Tc-MYBPA*-pGEM plasmid were loaded on the last lane as a positive control for the *Tc-MYBPA* primer set and as a negative control for the *AtUbiquitin* primer set. C, PA levels in mature seeds of plants shown in (**a**). PA levels were determined by extraction and DMACA reaction using procyanidin B2 as a standard. All the data are presented as means ± SE, n = 3. **P < 0.01 versus *tt2*; ***P < 0.001 versus *tt2*. FW, fresh weight

Based on the very high degree of sequence conservation with Arabidopsis TT2 (see above) we hypothesized that the candidate gene *Tc-MYBPA* encodes a protein transcription factor that participates in the regulation of the PA biosynthesis genes *LAR*, *ANR* and *LDOX*. To test this hypothesis, a genetic complementation test was performed by introduction of a constitutively expressed *Tc-MYCPA* coding sequence into the Arabidopsis *tt2* mutant [10], creating *Tc-MYBPA-tt2* transgenic plants. Twenty one hygromycin-resistant transgenic T1 plants were generated and all of them developed a normal phenotype regarding general plant health, vigor, size and height. Three independent hygromycin-resistant transgenic T1 plants of *Tc-MYBPA-tt2* were selected because of their increased seed coat color by visual observation. After staining with dimethylaminocinnamaldehyde (DMACA), a dye that can specifically interact with PAs and present a blue reaction product [41], 2 lines (Line 6 and Line 12) stained blue with DMACA (Fig. 7a), suggesting deposition of PAs in the seed coat. The other lines that did not develop an increased seed coat color also did not stain blue with DMACA (data not shown). In Line 6, the DMACA staining resulted in nearly the same intense color as in Col-0; while in line 12, the blue color was less intense than in Col-0, suggesting decreased PA levels compared to wild-type. RT-PCR using RNA extracted from T2 seedlings confirmed expression of the *Tc-MYBPA* gene in these transgenic lines and indicated that Line 6 had the highest expression level, which correlated with the highest PA levels as suggested by DMACA staining (Fig. 7b). PA levels in the two *Tc-MYBPA-tt2* lines were 2-8-fold higher than in the *tt2* background (Fig. 7c). *Tc-MYBPA-tt2* line 6, which had the highest *Tc-MYBPA* expression, had nearly the same PA concentration as in the Col-0 seeds. In the young seedlings, two transgenic lines (Line 6 and Line 12) accumulated elevated levels of anthocyanins in the hypocotyls compared to *tt2* mutant plants. Line 6, which has the highest *Tc-MYBPA* gene expression level, accumulated the most red/purple anthocyanin pigments.

**Fig. 8:**
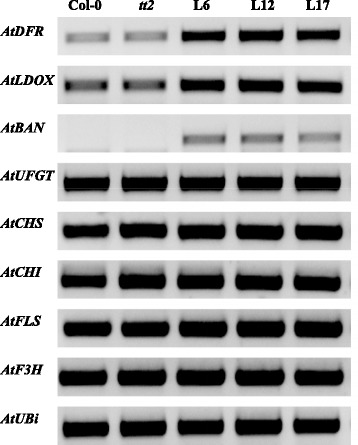
Semi-quantitative RT-PCR analysis of expression of flavonoid structural genes in young seedlings of the same *Arabidopsis* lines analyzed in Fig. 6. *DFR*, dihydroflavonol reductase; *LDOX*, leucoanthocyanidin dioxygenase; *BAN*, banyuls (anthocyanidin reductase); *UFGT*, UDP-Glc flavonoid glucosyltransferase; *CHS*, chalcone synthase; *CHI*, chalcone isomerase; *F3H*, flavonoid 3’-hydroxylase; *FLS*, flavonol synthase, *UBi*, Ubiquitin

In order to confirm that *Tc-MYBPA* activates PA synthesis genes, we used semi-quantitative RT-PCR to examine the expression of relevant genes in young seedlings of transgenic *Tc-MYBPA-tt2* lines, untransformed *tt2* mutant and wild-type plants (Fig. 8). Expression levels were measured for the PA-related structural genes (*DFR*, *LDOX* and *BAN*) as well as the general flavonoid pathway genes (chalcone synthase, *CHS*; chalcone isomerase, *CHI*; and flavonoid 3'-hydroxylase, *F3H*), a flavonol-specific gene (flavonol synthase; *FLS*) and an anthocyanin-specific gene (UDP-Glc-flavonoid glucosyltransferase, *UFGT*). Gene expression of *DFR* and *LDOX* was at about the same level as in the wild-type (Col-0) control and the *tt2* mutant, a result consistent with their contribution to anthocyanidin synthesis. In all transgenic lines, overexpression of *Tc-MYBPA* was found to activate the flavonoid late biosynthesis genes [10] related to PA synthesis (*DFR*, *LDOX* and *BAN*). There was a 2-fold increase of *DFR* gene expression in all transgenic lines, and an approximate 1.5-1.7-fold increase of *LDOX* gene expression. *BAN* was not expressed in either *tt2* or Col-0 seedlings but it was significantly activated in the transgenic lines, suggesting that *Tc-MYBPA* controls its activation. However, no significant gene activation was detected for all the other flavonoid genes including *CHS*, *CHI*, *F3H* representing the general flavonoid pathway, *FLS* representing the flavonol-specific pathway and *UFGT* representing the anthocyanin-specific pathway.

## Discussion

In this study, amino acid sequence motifs specific to the PA-regulating clade of MYB transcription factors from other species were used to identify a candidate cacao ortholog. We compared five genes from four species including Arabidopsis and *Lotus* TT2 [10, 20], grape VvMYBPA1 and VVMYBPA2 [23, 24] and poplar MYB134 [22]. Each of these has been experimentally demonstrated to play a key role in regulating the transcription of PA biosynthesis genes. Arabidopsis and *Lotus* TT2, poplar MYB134 and grape VvMYBPA2 formed a phylogenetic cluster with ZmC1 from maize, which has been shown to activate the Arabidopsis *ANR* promoter [10]. However, cacao *Tc-MYBPA* and grape VvMYBPA1 are not in the clade that contains most of the PA-regulating MYBs; they formed another cluster that is significantly closer to the TT2/C1 clade than to other functionally unrelated MYB regulators. By contrast, the multiple protein sequence alignment including all the known PA and anthocyanin-regulatory MYB proteins revealed some PA specific motifs in the N-terminal domain. Five sites (1 or 2 amino acids) were conserved in all PA-specific MYBs, including ZmC1, but were absent from all other anthocyanin-specific MYBs. The discrepancy between the phylogenetic analysis, which showed a separate clade of *Tc-MYBPA* and VvMYBPA1 distinct from all other PA-regulatory MYBs, and the protein alignment, which clearly showed highly conserved PA-specific protein motifs in all PA MYBs, may result from the low homology C-terminal domain of those R2R3 MYB proteins. Similar to the results of Bogs et al. [23], none of the conserved motifs in the C-terminal domain described by Stracke et al. [19] were found. By contrast, phylogenic analysis seems to be a strong predictor of the anthocyanin regulatory MYB proteins, with all the functionally proven anthocyanin specific MYB transcription factors falling into the same subgroup [15, 42–44]. Interestingly, grape and cacao also share the distinction, together with tea, of being commercial species containing the highest levels of PA in all commonly consumed foods [45].

The analysis of PA levels during leaf development revealed that PA synthesis in cacao leaves occurs at higher levels in young leaves then in older leaves. This correlates with the synthesis of anthocyanins, which are present at a much higher concentrations in younger stage leaves than in mature leaves [46]. Anthocyanin and PA synthesis share common structural enzymes in the PA synthesis pathway, including anthocyanin synthase (ANS/LDOX), which produces cyanidins used in the ANR reaction leading to epicatechins and in the UFGT reaction leading to anthocyanidins. Consistent with the PA and anthocyanin accumulation patterns, the cacao PA-specific structural genes *ANR* and *LAR* and the anthocyanin PA-common gene *ANS* were all co-regulated in developing leaves and more highly expressed in younger leaves compared to older leaves. Expression of the *Tc-MYBPA* gene correlated well with PA accumulation rates and expression of the PA biosynthetic genes *TcANR*, *TcANS* and *TcANR*. Similar results were observed from *Tc-MYBPA* transcript profiling in young pods and exocarp tissues, in which *Tc-MYBPA* exhibits the exactly same pattern with the co-regulated PA synthesis genes *TcANR*, *TcANS* and *TcANR*, suggesting that the *Tc-MYBPA* protein is involved in regulation of PA biosynthesis in leaves, young pods and exocarp.

In cacao reproductive tissues, PA synthesis began in developing flowers prior to pollination and continued in fruits until maturation, while anthocyanin synthesis began at the onset of fruit ripening and paralleled PA synthesis until maturation. Distinct from co-regulated expression of *TcANS, TcANR* and *TcLAR* genes in fruit exocarp, the *TcANS* gene had a different expression pattern from that of *TcANR* and *TcLAR* in ovules. *TcANR* and *TcLAR* were still co-regulated in ovules throughout developmental stages and both dropped at 16 WAP when fruit ripening commences and anthocyanin synthesis begins, while *TcANS* expression remained relatively high at 16 WAP, likely contributing to anthocyanin synthesis. Surprisingly, *Tc-MYBPA* shared the same expression pattern with *TcANS* rather than with the PA-specific genes *TcANR* and *TcLAR*, and the expression level remained stable, showing no decrease at 16 WAP. Similar observations were observed regarding the expression pattern of *VvMYBPA1* in grape skins, in which *VvMYBPA1* retained a relatively high transcript level two weeks after the onset of ripening and PA synthesis completely stopped when anthocyanin synthesis began [23]. One interpretation is that the high levels of *VvMYBPA1* could also contribute to anthocyanin synthesis, as it could activate the promoter of the *VvANS* (*VvLDOX*) gene. Overall, the expression pattern of *Tc-MYBPA* suggests that the encoded protein is involved in regulation of PA biosynthesis; moreover, it may also be involved in regulation of anthocyanin biosynthesis.

Overexpression of *Tc-MYBPA* in the Arabidopsis *tt2* mutant complemented the PA-deficient phenotype in Arabidopsis mature seeds (Fig. 6). This indicated that this R2R3-type MYB transcription factor was able to substitute for the function of the key Arabidopsis PA regulator TT2. In contrast to grape VvMYBPA1 (the MYB protein most similar to *Tc-MYBPA*1), which can induce ectopic PA accumulation when overexpressed in Arabidopsis, *Tc-MYBPA-tt2* transgenic plants accumulated PAs only in the seed coat. This tissue specific phenotype was similar to Arabidopsis *TT2*, which also failed to induce PA accumulation in tissues other than seed coat when ectopically expressed. Gene expression analysis of *Tc-MYBPA-tt2* transgenic plants showed that overexpression of *Tc-MYBPA* induced only late flavonoid biosynthetic genes, *DFR, LDOX* and *BAN,* similar to Arabidopsis TT2, which also controls only the late flavonoid biosynthetic genes *DFR* and *BAN* [10]. By contrast, VvMYBPA1 regulates the entire flavonoid pathway branch leading to PA synthesis, including both early and late flavonoid biosynthetic genes [23].

In transgenic Arabidopsis expressing the *Tc-MYBPA* gene, an increased accumulation of anthocyanins was also observed in hypocotyls of young seedlings; especially in Line 6, which showed an obvious visual color difference compared to untransformed controls. This could be explained by the ability of *Tc-MYBPA* to induce the expression of *LDOX (ANS*), which is a structural gene contributing to both the anthocyanin and the proanthocyanin pathway. This is different from the Arabidopsis TT2 MYB transcription factor, which has been shown to involved specifically in the genetic control of flavonoid late biosynthesis genes (LBGs) including *DFR*, *LDOX* and *BAN* only in seeds [10]. However, both *BAN* and *TT2* are not expressed in seedlings, while both *DFR* and *LDOX* are expressed in seedlings, contributing to anthocyanin synthesis. Their expression is controlled by another MYB transcription factor, AtPAP1 [47–49], whereas over-expression of AtTT2 did not increase the expression levels of LBGs in seedlings, with the exception of *BAN*, suggesting its specific involvement in PA synthesis [10]. The activity of *Tc-MYBPA* was in contrast to grape VvMYBPA1. Although VvMYBPA1 could activate the *VvLDOX* gene promoter in transient reporter gene assays, it failed to induce anthocyanin synthesis when overexpressed in Arabidopsis [23]. Bogs et al. also showed that anthocyanin synthesis in grape was regulated by another MYB transcription factor VvMYBA2 [50]. However, the data from this research in transgenic Arabidopsis demonstrated that activation of anthocyanin synthesis was consistent with the *Tc-MYBPA* gene expression pattern in cacao, which was co-regulated with the *TcANS* gene and coincided with anthocyanin synthesis. Taken together, in cacao, *Tc-MYBPA* appeared to be capable of regulating both the PA and anthocyanin pathway by activating late PA biosynthetic genes. Potentially, this could provide a means to manipulate the amount and composition of PAs and anthocyanin together in cacao and possibly in other fruits. The different activities of the related MYB transcription factor genes from diverse species could reflect the evolutionary specialization of duplicated gene family members which appears to have taken slightly different functions over evolutionary time and can account in part for the differences in PA and anthocyanin accumulation patterns in these species.

## Conclusion

In summary, our results support the conclusion that *Tc-MYBPA* from cacao is involved in regulation of transcription of several PA biosynthesis genes. This is based on several lines of evidence. First, protein sequence comparison showed that *Tc-MYBPA* was most similar to the grape PA transcriptional regulator VvMYBPA1 and shared the conserved motifs of all the other functionally characterized R2R3-MYB PA synthesis regulators. Second, transcript profiling showed that *Tc-MYBPA* was expressed in all tissues accumulating PAs and consistently co-regulated with PA biosynthesis structural genes including *TcANR, TcANS* and *TcLAR*. Third, over-expression of *Tc-MYBPA* in Arabidopsis was able to functionally complement the PA-deficient phenotype in the seeds of the *tt2* mutant and resulted in a significant increase of PA accumulation compared to the *tt2* mutant. This was the result of activation of the PA biosynthetic genes including *DFR, LDOX* and *ANR* as shown by gene expression analysis of transgenic plants relative to untransformed *tt2* and Col-0 plants.

## Methods

### Plant material

Two *Theobroma cacao* varieties: Scavina 6 and Amelonado were used for this study. Cacao plants were grown in greenhouse as previously described [51]. Leaf and flower tissues were collected from Scavina 6 plants. For leaf tissues, various stage leaves were collected. The definition of leaves stages were previously described [52], briefly, Stage A leaves are newly emerged and are 5–10 cm long; stage B leaves are larger, soft, red and translucent, 10–15 cm long; Stage C leaves are green and remain soft; Stage D leaves are at an early stage of lignification; Stage E leaves are fully lignified and mature. Stage A and B leaves were pooled together because of the limited amount of Stage A leaves. Cacao pods were obtained by hand pollinating Amelonado (a self-compatible variety). Upon harvesting, pods were bisected, and seeds and pod exocarps collected separately. Exocarp samples represent the outer 1–3 mm layer of the fruit obtained using a fruit peeler. All samples were frozen in liquid nitrogen upon collection and stored at −80 °C until extraction.

Arabidopsis plants (*Arabidopsis thaliana*) were grown in soil at 22 °C, 50 % humidity and a 16 h/8 h light/dark photoperiod in a growth chamber (Conviron, Pembina, ND, USA). Plants grown aseptically were plated on MS medium [53] with 2 % (w/v) sucrose solidified with 0.6 % (w/v) agar. Arabidopsis ecotype Columbia (Col-0) plants were used as the wild type. T-DNA insertion mutant tt2 (SALK_005260) were obtained from The Arabidopsis Biological Resource Center (Columbus, OH, USA).

### Isolation of a *Tc-MYBPA* cDNA from *Theobroma cacao*

Total RNA from stage A/B leaves of *Theobroma cacao* (Scavina 6) was isolated using a modified cetyl trimethyl ammonium bromide (CTAB) extraction method as previously described [54] with the following modifications. RNA isolated from the CTAB extraction and LiCl precipitation was further purified and concentrated using RNeasy columns (Qiagen, Valencia, CA, USA), but the phenol/chloroform extraction and sodium acetate/ehanol precipitation steps were omitted. The quality of RNA was verified by observing absorbance ratios of A260/A280 (1.8-2.0) and A260/A230 (1.8-2.2) and by separating 200 ng RNA samples on 0.8 % agarose gels to examine intact ribosomal bands.

First strand cDNA was synthesized using the SMART RACE cDNA amplification kit (Clontech, Mountain View, CA, USA). The putative EST sequence of *Tc-MYBPA* was obtained by searching the *Theobroma cacao* EST database (http://esttik.cirad.fr/) [34] using BLAST (program: tBLASTn) [55] with the protein sequence of *TT2* (AT5G35550) from *Arabidopsis* thaliana as the query sequence. The ORF of putative *Tc-MYBPA* was amplified with the Advantage cDNA PCR Kit (Clontech, Mountain View, CA, USA) using cDNA from stage A/B leaves as template with the following primer pairs: *Tc-MYBPA*_F (5’- GT*CCATGG*GAAGGGCTCCTTGTTGTTC -3’) and *Tc-MYBPA*_R (5’- A*GCGGCCGC*TCAGATCAATAATGATTCAGC -3’). To facilitate the subsequent cloning into binary vectors, an *Nco*I site (CCATGG) was added at the start codon (ATG) and a *Not*I site (GCGGCCGC) was added immediately 3' to the stop codon (TCA) respectively (sites are shown in italics and the start or stop codons are underlined). The PCR reaction was carried out in a total volume of 20 μL at 94 °C for 5 min; 5 cycles of 94 °C for 30 s, 55 °C for 30 s, and 72 °C for 1 min; another 23 cycles of 94 °C for 30 s, 60 °C for 30 s, and 72 °C for 1 min; followed by a final extension at 72 °C for 5 min. The PCR products were gel purified and cloned into the pGEM-T Easy plasmid (Promega, Madison, WI, USA) and replicated in *E. coli* strain DH5α. DNA sequencing was performed using 12 of the resulting DNA clones (pGEMT-*Tc-MYBPA*), and two clones had the precise sequence of the consensus sequences. One clone (pGEMT-*Tc-MYBPA*-3) was chosen for cloning into the binary vector for plant transformation and subsequent experiments.

### Protein sequence alignment and phylogenetic analysis

PA-specific R2R3-MYB protein sequences were retrieved from GenBank (http://www.ncbi.nlm.nih.gov/Genbank/), including AtTT2 from Arabidopsis (CAC40021) [10], VvMYBPA1 and VvMYBPA2 from grape (AM259485, ACK56131) [23, 24], LjTT2a, LjTT2b and LjTT2c from *Lotus japonicus* (AB300033, AB300034, AB300035) [21] and MYB134 from *Populus tremuloides* (FJ573151) [22]. A protein sequence alignment performed with the ClustalW algorithm was used to construct a phylogenetic tree using the neighbor-joining method in the MEGA package [56]. One thousand bootstrap datasets were used to estimate the confidence of each tree clade. Protein sequence alignment of anthocyanin- and proanthocyanin-specific MYB proteins was performed using the same method as was for the phylogenetic tree but was edited and displayed using GENEDOC software (Version 2.6.02, http://www.nrbsc.org/gfx/genedoc/gddl.htm).

### Proanthocyanidin (PAs) quantification

To extract soluble PAs from cacao tissues, 0.3-0.5 g of frozen tissues were ground into a fine powder in liquid nitrogen and then extracted with 5 mL of extraction solution (70 % acetone: 29.5 % water: 0.5 % acetic acid) by vortexing for 5 s followed by water bath sonication for 15 min using a bench top ultrasonic cleaner (Model 2510, Bransonic, Danbury, CT, USA). To extract soluble PAs from Arabidopsis seeds, the same extraction solution and method were applied, except that 100-500 mg dry seeds were used as grinding samples, and 500 μL extraction solution were used. After sonication, samples were vortexed again and centrifuged at 2500 g for 10 min. The supernatant was transferred to a new tube and the pellet was re-extracted twice as above. Pooled supernatants were extracted twice with hexane to remove fat and chlorophyll and then filtered through a 0.45 μm polytetrafluoroethylene (PTFE) syringe filter (Millipore, Billerica, MA, USA). Depending on availability of plant samples, different numbers of biological replicates were performed for cacao and Arabidopsis samples. For cacao, there were at least five biological replicates, and for Arabidopsis, there were three biological replicates.

To quantify PA levels, 50 μL aliquots of samples were mixed with 200 μL of dimethylaminocinnamaldehyde (DMACA; Sigma-Aldrich, MO, USA) reagent (0.1 % DMACA, 90 % reagent-grade ethanol, 10 % HCl) in 96-well microtiter plates. Absorption was measured at 640 nm at one-minute intervals for 20 min, and the mean value of peak readings during this time period was recorded. For each biological replicate, triple technical replicates were performed to obtain mean values. The total PA levels were calculated using a standard molar absorbance curve prepared using procyanidin B2 (Indofine, NJ, USA).

For quantitative analysis of insoluble PAs from cacao tissues, the residues from soluble PA extractions were air dried in an exhaust hood for two days, weighed, and 5 mL butanol-HCl reagent (95 % butan-1-ol: 5 % concentrated HCl) was added and the mixture was sonicated for one hour followed by centrifugation at 2500 g for 10 min. An aliquot of clear supernatant was diluted 40-fold in butanol-HCl reagent and absorbance was measured at 550 nm to determine the amount of background absorption. The samples were then boiled for 1 h with vortexing every 20 min, cooled to room temperature and centrifuged again at 2500 g for 10 min. The supernatant from boiled sample was diluted 40-fold in butanol-HCl reagent and absorbance was measured at 550 nm. The values were normalized by subtraction of the background absorbance and the PA levels were calculated as cyanidin equivalents using cyanidin-3-glucoside (Sigma-Aldrich, MO, USA) as standards.

To visualize the presence of PAs in Arabidopsis young seedlings and dry seeds, tissues were immersed in 4-dimethylaminocinnamaldehyde (DMACA) reagent (2 % (w/v) DMACA, 90 % ethanol, 10 % HCl) for 2 days as described previously [9] and then washed 3 times with 70 % ethanol.

### Transformation of Arabidopsis

The coding sequence of *Tc-MYBPA* was excised from the intermediate cloning vector (pGEMT-*Tc-MYBPA*-3) with *Nco*I and *Not*I restriction enzymes and introduced into the pE2113-EGFP [51] intermediate vector to substitute the coding sequence of *Tc-MYBPA* for the original EGFP coding sequence. As a result, the cacao gene coding sequence is located immediately downstream of the very strong E12-Ω promoter (a modified CaMV35S promoter) and upstream of the CaMV35S terminator. The over-expression cassette was excised out from pE2113 vector with *Ecor*I and *Pvu*II restriction enzymes and introduced into the pCAMBIA-1300 binary vector (CAMBIA, Canberra, Australia).

This binary transformation construct was introduced into *Agrobacterium tumefaciens* strain AGL1 [57] by electroporation as previously described [58]. Arabidopsis transformation was carried out using the floral dip method [59], and T1 transgenic plants were selected on MS media supplemented with 2 % sucrose, 0.65 % agar and 25 mg/L hygromycin. Hygromycin-resistant T1 seedlings were transferred to soil 7 days after germination and grown in a growth chamber as described above.

### Gene expression analysis

Total RNA from leaves, flowers, pods, pod exocarp and ovules of *Theobroma cacao* (Scavina 6 and Amelonado) was isolated as described above. Total RNA from young Arabidopsis seedlings was isolated using the RNeasy Plant mini kit (Qiagen, Valencia, CA, USA). cDNA was synthesized from 1 μg of total RNA in a total volume of 20 μL using M-MuLV Reverse Transcriptase (NEB, Ipswich, MA, USA) according to the supplier’s protocols, and 2 μL of this reaction were used in the subsequent RT-PCR reactions.

Semi-quantitative RT-PCR was performed to measure gene expression levels as previously described [60] with the following modifications: The primers for Arabidopsis cDNA span two exons, giving products of about 500 bp, and thus are mRNA specific, avoiding potential amplification from genomic DNA contamination. The primers sets used are listed in Table 1 below.

**Table 1:** Sequences of the primers used in the gene expression study

Gene	Protein	Locus ID	Primer	Sequence (5’ to 3’)
ANS (LDOX)	leucoanthocyanidin dioxygenase	Tc03g026420	TcANS_RTF	ACCTTGTTAACCATGGGATCTCGG
			TcANS_RTR	GACGGTGTCACCAATGTGCATGAT
ANR	anthocyanidin reductase	Tc06g018030	TcANR_RTF	TGCTTGAGAAGGGCTACGCTGTTA
			TcANR_RTR	AAAGATGTGGCAAGGCCAATGCTG
LAR	Leucoanthocyanidin reductase	Tc03g002450	TcLAR_RTF	AATTCCATTGCAGCTTGGCCCTAC
			TcLAR_RTR	GGCTTGCTCACTGCTTTGGCATTA
UB	polyubiquitin	Tc06g018641	TcUb_RTF	AGCTGAGAGATTCCGTTGTCCAGA
			TcUb_RTR	CCCACATCAACCAGACTTTGAGTTC
MybPA	TT2 like MYB transcriptoin factor	Tc01g034240	Tc-MYBPA_RTF	GATGGGAAGGGCTCCTTGTTG
			Tc-MYBPA_RTR	ATCTCGTTATCGGTTGGACCAG
DFR	dihydroflavonol 4-reductase	AT5G42800	AtDFR_RTF	CCGTGTGTGTAACCGGCGCT
			AtDFR_RTR	TGCGCCTCGTTCCGAGTGAT
LDOX (ANS)	leucoanthocyanidin dioxygenase	AT4G22880	AtLDOX_RTF	CACCAAGTGATTACATAGAAGCAACG
			AtLDOX_RTR	TCACCATCTCCGGCAACGGC
BAN (ANR)	anthocyanidin reductase	AT1G61720	AtBAN_RTF	TGGTGGCACGGGAAACTTAGCC
			AtBAN_RTR	CGGTCACATGCATTTCTTTCCCGGT
UFGT	UDP-glucosyl transferase	AT5G17050	AtUFGT_RTF	ACCATCGGTGTCAAAGAAGTAGGTG
			AtUFGT_RTR	GCGGTGCCCATGGAACCACT
CHS	chalcone synthase	AT5G13930	AtCHS_RTF	TCAAGCGCATGTGCGACAAGT
			AtCHS_RTR	CGGCGGCGCCATCACTGAAA
CHI	chalcone isomerase	AT3G55120	AtCHI_RTF	TCATCCAACGCCTGCGCCTC
			AtCHI_RTR	ACGCAACCGTAAGAGAGCCGGT
F3H	flavanone 3-hydroxylase	AT3G51240	AtF3H_RTF	ACCTCCAGGGAGAGGCTGTGC
			AtF3H_RTR	AAATGGCCGTGGTCGCCGAG
FLS	flavonol synthase 1	AT5G08640	AtFLS_RTF	ATCTGGCCACCGTCATGCGTC
			AtFLS_RTR	CCTCCCATTACTCAACCTCAGAATC

To ensure accurate semi-quantitative RT-PCR measurements, each primer set was tested in time course PCR reactions to measure amplification kinetics and to determine the optimal PCR cycle in which the reaction is well within the linear range (28 cycles). PCR reactions were carried out in a total volume of 20 μL at 94 °C for 5 min; 28 cycles of 94 °C for 30 s, 55 °C for 30 s, and 72 °C for 45 s; followed by a final extension at 72 °C for 5 min. The PCR products were visualized on 1 % agarose gels stained with ethidium bromide and photographed using a Molecular Imager Gel Doc XR+ System equipped with a 16-bit CCD camera (Bio-Rad Laboratories, Hercules, CA). Relative fluorescent intensity of the separated PCR products was quantified using Quantity One 1-D Analysis Software (Bio-Rad Laboratories, Hercules, CA). Expression levels were calculated relative to the expression of *TcActin* in each sample.

### Availability of supporting data

The phylogenetic tree for the study have been submitted to DRYAD (doi:10.5061/dryad.57fc0).

## Additional file

Additional file 1: Figure S1. Sequence analysis of other putative cacao TT2 like genes. **Figure S2.** MYB-binding motifs in the promoters of cacao PA synthesis genes, *TcANR, TcLAR*, *TcAN*S, transcription factor *TcMybPA* and Arabidopsis *AtTT2* detected using the PLACE database (http://www.dna.affrc.go.jp/PLACE/signalup.html).

## Abbreviations

PAs: Proanthocyanidins
*DFR*: Dihydroflavanol reductase
ANS: Anthocyanin synthase
*LDOX*: Leucoanthocyanidin dioxygenase
*ANR*: Anthocyanidin reductase
ORFs: Open reading frames
ESTs: Expressed sequence tags
DMACA: Dimethylaminocinnamaldehyde
WAP: Week after pollination
