# Supplementary material for: Tc-MYBPA an Arabidopsis TT2-like transcription factor and functions in the regulation of proanthocyanidin synthesis in Theobroma cacao
Source: BMC Plant Biol. 2015 Jun 25;15:160. doi: 10.1186/s12870-015-0529-y (PMC4481123; doi:10.1186/s12870-015-0529-y)
Supplement: Additional file 1: Figure S1. — Sequence analysis of other putative cacao TT2 like genes. Figure S2. MYB-binding motifs in the promoters of cacao PA synthesis genes, TcANR, TcLAR, TcANS, transcription factor TcMybPA and Arabidopsis AtTT2 detected using the PLACE database (http://www.dna.affrc.go.jp/PLACE/signalup.html). [file 12870_2015_529_MOESM1_ESM.pdf]

Blast results by querying AtTT2 to cacao genome

Sequences producing significant alignments:	(bits)	Value
Tc00_g016740 GHMYB10	199	2e-51
Tc09_g007160 MybPA2	194	9e-50
Tc09_g005570 Putative GHMYB10	191	4e-49
Tc09_g005580 Putative Transcription factor MYB558	191	6e-49
Tc01_g029340 Putative uncharacterized protein	189	1e-48
Tc01_g004300 Putative MybPA2	189	3e-48
Tc02_g026610 Putative uncharacterized protein	188	3e-48
Tc01_g034240 TT2 like MYB transcriptoin factor	168	5e-42
Tc09_g005590 Putative uncharacterized protein	167	6e-42
Tc02_g027560 Transcription repressor MYB4	167	1e-41

```
>Tc00_t016740
MGRNPCCSKEGLNRGAWTAHEDKILTQYIKAHGEGSWRNLPKRAGLKRCGKSCRLRWLNYLRPDIKRGNISADEEELIIRLH
KLLGNRWSLIAGRLPGRTDNEIKNYWNTNLGKKVQHHQNPSAAPGHNKSNGRVTRKREAQLATAAGTPSSHVVRTRAIRCSK
VFINPHQKIEPFDRNREAKPSIDGECRPMTQTSAPSPIKALSENEDQNVHNKPFDFMVDFNMGEFCLSELLNSDFSDLVGLN
YSNDSSNAISPSSDQPLIFSEEILQDWTSSHHAQQNVASNPHSLASFLDYGEEWLAE
```

alignment with top EST contig hit

>CL1309Contig1

```
          Length = 1392

 Score =  202 bits (515), Expect = 2e-52,   Method: Compositional matrix adjust.
 Identities = 90/116 (77%), Positives = 104/116 (89%)
 Frame = -1

Query: 1    MGRNPCCSKEGLNRGAWTAHEDKILTQYIKAHGEGSWRNLPKRAGLKRCGKSCRLRWLNY 60
            MGR PCCSK GL+RG WT  ED +L +YI+AHG+G WR+LPK+AGL RCGKSCRLRW+NY
Sbjct: 1167 MGRAPCCSKVGLHRGPWTPREDTLLVKYIQAHGDGHWRSLPKKAGLLRCGKSCRLRWMNY 988

Query: 61   LRPDIKRGNISADEEELIIRLHKLLGNRWSLIAGRLPGRTDNEIKNYWNTNLGKKV 116
            LRPDIKRGNI+ DE++LIIRLH LLGNRWSLIAGRLPGRTDNEIKNYWNT+L K++
Sbjct: 987  LRPDIKRGNITPDEDDLIIRLHSLLGNRWSLIAGRLPGRTDNEIKNYWNTHLSKRL 820
```

```
>Tc09_t007160
MGRKPCCAKVGLNRGAWSAREDKILTNYIKIHGEGKWRDLPQRAGLKRCGKSCRLRWLNYLRPDIKRGNISSEEEELIVRLH
KLLGNRWSLIAGRLPGRTDNEIKNYWNTNLSKRVQGNKNVTPKGTQTSNMQLNKLRKLGCIKSSQMEITTAEHKVIRTKAVK
CTKAVNVNVPHLSDCQTAENNSVPKSESTTASESPSSSAINEDNSMDFLIDFDINELLVSDAPMSDLHQTQQVDENGENVVD
NWECRNGFNLNIDDVELYSSVDELARPDNWRPSTDLFQTSETLDLLSLASYLNLEN
```

alignment with top EST contig hit

>CL1309Contig1

```
          Length = 1392

 Score =  211 bits (538), Expect = 4e-55,   Method: Compositional matrix adjust.
 Identities = 96/135 (71%), Positives = 112/135 (82%)
 Frame = -1

Query: 1    MGRRPCCSKEGMNRGAWTLTEDKILTDYVKVHGEGKWRNIPKEAGLKRCGKSCRLRWMNY 60
            MGR PCCSK G++RG WT  ED +L  Y++ HG+G WR++PK+AGL RCGKSCRLRWMNY
Sbjct: 1167 MGRAPCCSKVGLHRGPWTPREDTLLVKYIQAHGDGHWRSLPKKAGLLRCGKSCRLRWMNY 988

Query: 61   LRPDIKRGNISPDEEDLIIRLHKLLGNRWSLIAGRLPGRTDNEIKNYWNTHLSKRGQGKE 120
            LRPDIKRGNI+PDE+DLIIRLH LLGNRWSLIAGRLPGRTDNEIKNYWNTHLSKR   +
Sbjct: 987  LRPDIKRGNITPDEDDLIIRLHSLLGNRWSLIAGRLPGRTDNEIKNYWNTHLSKRLLSQG 808

Query: 121  RGPKSNHSKVGKKPI 135
```

```
            P + H K+ + P+
Sbjct: 807  TDPNT-HKKLSEPPV 766
```

>Tc09_t005570
MGRRPCCSKEGMNRGAWTLTEDKILTDYVKVHGEGKWRNIPKEAGLKRCGKSCRLRWMNYLRPDIKRGNISPDEEDLIIRLH
KLLGNRWSLIAGRLPGRTDNEIKNYWNTHLSKRGQGKERGPKSNHSKVGKKPITVKVDEDLVSSRVIRTKALRFKEVFITPD
AQQHRIENHHRNVAIPPSMDPNLVQNVPAGSESSDRSLITLSSNEEYPVDFMVDISAGEISVPEIFASDFTSFSHDLGASEM
DKVMRGDDADNYMSKIAPSQAFLLPEEMEQNWSGSDYYIQANLDSDFGSLTAFLESAEEWLI

alignment with top EST contig hit

```
>CL1309Contig1
          Length = 1392

 Score =  211 bits (538), Expect = 4e-55,   Method: Compositional matrix adjust.
 Identities = 96/135 (71%), Positives = 112/135 (82%)
 Frame = -1

Query: 1    MGRRPCCSKEGMNRGAWTLTEDKILTDYVKVHGEGKWRNIPKEAGLKRCGKSCRLRWMNY 60
            MGR PCCSK G++RG WT  ED +L  Y++ HG+G WR++PK+AGL RCGKSCRLRWMNY
Sbjct: 1167 MGRAPCCSKVGLHRGPWTPREDTLLVKYIQAHGDGHWRSLPKKAGLLRCGKSCRLRWMNY 988

Query: 61   LRPDIKRGNISPDEEDLIIRLHKLLGNRWSLIAGRLPGRTDNEIKNYWNTHLSKRGQGKE 120
            LRPDIKRGNI+PDE+DLIIRLH LLGNRWSLIAGRLPGRTDNEIKNYWNTHLSKR   +
Sbjct: 987  LRPDIKRGNITPDEDDLIIRLHSLLGNRWSLIAGRLPGRTDNEIKNYWNTHLSKRLLSQG 808

Query: 121  RGPKSNHSKVGKKPI 135
              P + H K+ + P+
Sbjct: 807  TDPNT-HKKLSEPPV 766
```

>Tc09_t005580
MGRKPCCSKEGLNRGAWSAIEDQILIDYIKTNGEGKWGNIPKKAGLKRCGKSCRLRWLNYLRPDIKRGNISQEEEDLIIRLH
KLLGNRWSLIAGRIPGRTDNEIKNYWNSTLGKKVKAEQAKQSKDDLKKAKIQPLAETPQSTALRTKASKCSTCLVTQEAYKA
EIIDADLVHDARLEPPKSEGTLLSTISEEAHSVNSALEFDIGELLASDISDSEFWNCCQVNNTSMEEAESSGVARNMWLSSE
QHLQFSEEMLTDWIRDDI

alignment with the top contig hit

```
>CL2621Contig1
          Length = 1332

 Score =  201 bits (511), Expect = 4e-52,   Method: Compositional matrix adjust.
 Identities = 98/185 (52%), Positives = 128/185 (69%), Gaps = 15/185 (8%)
 Frame = -1

Query: 3    RKPCCSKEGLNRGAWSAIEDQILIDYIKTNGEGKWGNIPKKAGLKRCGKSCRLRWLNYLR 62
            RKPCC K+G N+GAWS  EDQ LIDYI+T+GEG W ++PK AGL RCGKSCRLRW+NYLR
Sbjct: 1224 RKPCCDKQGTNKGAWSKQEDQKLIDYIRTHGEGCWRSLPKAAGLHRCGKSCRLRWINYLR 1045

Query: 63   PDIKRGNISQEEEDLIIRLHKLLGNRWSLIAGRIPGRTDNEIKNYWNSTLGKKVKAEQAK 122
            PDIKRGN +Q+EEDLII+LH LLGNRWSLIAGR+PGRTDNE+KNYWNS + +K+
Sbjct: 1044 PDIKRGNFAQDEEDLIIKLHALLGNRWSLIAGRLPGRTDNEVKNYWNSHIKRKLIKLGID 865

Query: 123  QSKDDLKKAKIQP--------------LAETP-QSTALRTKASKCSTCLVTQEAYKAEII 167
             +   L +  ++P              +A  P +S+A + + S  ++CL  + + +   +
Sbjct: 864  PNNHKLNQYPLRPQPQHVSPSSPASLNVARKPRRSSAAKDRVSDAASCLEDERSGRVSKL 685

Query: 168  DADLV 172
            D DL
Sbjct: 684  DLDLT 670
```

>Tc01_t029340
MWMRRVAGGNHRMFLIFGWQTGSKTSMLCSSFRTSIKTGNSDPIHWETTANFRLGVCTFSYIQTPAQNTGRDTIEQQQMGRK
PCCSKEEGLNRGAWTATEDKILTDYIKARGEGKWRSIPKAAGLKRCEKSCRLRWLNYLRPGIKRGNITRDEEDLIIRLHKLL
GNRWSLIAGRLPGRTDNEIKNYWNTVLSKRAQVKKFDHTNKDETKQRFISWSKKAPTSSGVIQAKAGRCTKVFTTPQQQVIG
RGENNNIARTAPSTDAKFVHETAVESGFSDGSFTLLSSKEENPSISKFAMDFDIGDINISEALASDFAQLGDFELRDINSVI
YEYGTNDCGQALLSAEGMVGNWSGNDCVEANLDSDFGFLAAFLGSAEL

alignment with the top EST contig hit

```
>CL1309Contig1
          Length = 1392

 Score =  196 bits (498), Expect = 2e-50,   Method: Compositional matrix adjust.
 Identities = 95/151 (62%), Positives = 111/151 (73%)
 Frame = -1

Query: 74   IEQQQMGRKPCCSKEEGLNRGAWTATEDKILTDYIKARGEGKWRSIPKAAGLKRCEKSCR 133
            + ++ MGR PCCSK  GL+RG WT  ED +L  YI+A G+G WRS+PK AGL RC KSCR
Sbjct: 1182 LRERGMGRAPCCSKV-GLHRGPWTPREDTLLVKYIQAHGDGHWRSLPKKAGLLRCGKSCR 1006

Query: 134  LRWLNYLRPGIKRGNITRDEEDLIIRLHKLLGNRWSLIAGRLPGRTDNEIKNYWNTVLSK 193
            LRW+NYLRP IKRGNIT DE+DLIIRLH LLGNRWSLIAGRLPGRTDNEIKNYWNT LSK
Sbjct: 1005 LRWMNYLRPDIKRGNITPDEDDLIIRLHSLLGNRWSLIAGRLPGRTDNEIKNYWNTHLSK 826

Query: 194  RAQVKKFDHTNKDETKQRFISWSKKAPTSSG 224
            R   +  D     +  +  +   +K  +S G
Sbjct: 825  RLLSQGTDPNTHKKLSEPPVQQVEKRKSSRG 733
```

>Tc01_t004300
MGRKPCCLKEGVNKGAWSAQEDKILTNYIELHGEGKWNTLPQKAGIRLNRCGKSCRLRWMNYLRPGIKRGNISPDEEDLIIR
LHRLLGNRWSLIAGRLPGRTDNEIKNYWNSVLSKKLNGDASKPPNRQCECEGKESVEKYRSGSTPNVIAPKARRCTGTLISL
VKGNHLPNNPCLASSTPADDELTLESQKDDVQMPNVEDEALIDSELYMPNTLDYLDFSQTRIEDMFENGNMHESELAPMLEM
ELENLTSLLGLEDEWRELDVEND

alignment with the top EST contig hit

```
>CL1309Contig1
          Length = 1392

 Score =  191 bits (486), Expect = 4e-49,   Method: Compositional matrix adjust.
 Identities = 88/125 (70%), Positives = 103/125 (82%)
 Frame = -1

Query: 1    MGRKPCCLKEGVNKGAWSAQEDKILTNYIELHGEGKWNTLPQKAGIRLNRCGKSCRLRWM 60
            MGR PCC K G+++G W+ +ED +L  YI+ HG+G W +LP+KAG  L RCGKSCRLRWM
Sbjct: 1167 MGRAPCCSKVGLHRGPWTPREDTLLVKYIQAHGDGHWRSLPKKAG--LLRCGKSCRLRWM 994

Query: 61   NYLRPGIKRGNISPDEEDLIIRLHRLLGNRWSLIAGRLPGRTDNEIKNYWNSVLSKKLNG 120
            NYLRP IKRGNI+PDE+DLIIRLH LLGNRWSLIAGRLPGRTDNEIKNYWN+ LSK+L
Sbjct: 993  NYLRPDIKRGNITPDEDDLIIRLHSLLGNRWSLIAGRLPGRTDNEIKNYWNTHLSKRLLS 814

Query: 121  DASKP 125
              + P
Sbjct: 813  QGTDP 799
```

>Tc02_t026610

```
MGRKPCCSQEEGLNRGTWTATEDKILTEYIKAHGEGKWRSIPKAAGLKRCGKSCRLRWLNYLRPDIKRGNITRDEEDLIIRL
HKLLGNRWSLIAGRLPGRTDNEIKNYWNSVLSKRAQVKEFDHTNKDEKKQRFISCSRKAPTSSGVIHAKAGRCTKVSFSPQQ
KVTGEDDNNTIVRTAPSMDVELVHDTAVVSGSSDGSFTLLSSKEENPSISRFAMDFDIGESSIPEALASDFSQLSDFDFCDI
NTVIYEYGTKDNGQALVSLEGMVGNWSGNGLVDANLDSFGFLAGFLESDGDLTI
```

alignment with the top EST contig hit

```
>CL1309Contig1
          Length = 1392

 Score =  202 bits (513), Expect = 3e-52,   Method: Compositional matrix adjust.
 Identities = 92/116 (79%), Positives = 102/116 (87%)
 Frame = -1

Query: 1    MGRKPCCSQEEGLNRGTWTATEDKILTEYIKAHGEGKWRSIPKAAGLKRCGKSCRLRWLN 60
            MGR PCCS+  GL+RG WT  ED +L +YI+AHG+G WRS+PK AGL RCGKSCRLRW+N
Sbjct: 1167 MGRAPCCSKV-GLHRGPWTPREDTLLVKYIQAHGDGHWRSLPKKAGLLRCGKSCRLRWMN 991

Query: 61   YLRPDIKRGNITRDEEDLIIRLHKLLGNRWSLIAGRLPGRTDNEIKNYWNSVLSKR 116
            YLRPDIKRGNIT DE+DLIIRLH LLGNRWSLIAGRLPGRTDNEIKNYWN+ LSKR
Sbjct: 990  YLRPDIKRGNITPDEDDLIIRLHSLLGNRWSLIAGRLPGRTDNEIKNYWNTHLSKR 823
```

```
>Tc01_t034240
MGRAPCCSKVGLHRGPWTPREDTLLVKYIQAHGDGHWRSLPKKAGLLRCGKSCRLRWMNYLRPDIKRGNITPDEDDLIIRLH
SLLGNRWSLIAGRLPGRTDNEIKNYWNTHLSKRLLSQGTDPNTHKKLSEPAVQQVKKRKSSRGNSNKKQNNSKGKGAKVEPE
KPKVHLPKPVRVTSFSLPRNDSFDQCNTFSTVSSSQGGEGGLGTEVVQGPWSDNVNDDENGTGFLAAYDDHGFVNGSDFECQ
SHVPASDDDNSLEKLYEEYLQLLKTNDDQVQLDSFAESLLI
```

alignment with the top EST contig hit

```
>CL1309Contig1
          Length = 1392

 Score =  514 bits (1325), Expect = e-146,   Method: Compositional matrix adjust.
 Identities = 252/287 (87%), Positives = 252/287 (87%)
 Frame = -1

Query: 1    MGRAPCCSKVGLHRGPWTPREDTLLVKYIQAHGDGHWRSLPKKAGLLRCGKSCRLRWMNY 60
            MGRAPCCSKVGLHRGPWTPREDTLLVKYIQAHGDGHWRSLPKKAGLLRCGKSCRLRWMNY
Sbjct: 1167 MGRAPCCSKVGLHRGPWTPREDTLLVKYIQAHGDGHWRSLPKKAGLLRCGKSCRLRWMNY 988

Query: 61   LRPDIKRGNITPDEDDLIIRLHSLLGNRWSLIAGRLPGRTDNEIKNYWNTHLSKRLLSQG 120
            LRPDIKRGNITPDEDDLIIRLHSLLGNRWSLIAGRLPGRTDNEIKNYWNTHLSKRLLSQG
Sbjct: 987  LRPDIKRGNITPDEDDLIIRLHSLLGNRWSLIAGRLPGRTDNEIKNYWNTHLSKRLLSQG 808

Query: 121  TDPNTHKKLSEPAVQQVXXXXXXXXXXXXXXXXXXXXXAKVEPEKPKVHLPKPVRVTSFS 180
            TDPNTHKKLSEP VQQV                     AKVEPEKPKVHLPKPVRVTSFS
Sbjct: 807  TDPNTHKKLSEPPVQQVEKRKSSRGNSNKKQNNSKGKGAKVEPEKPKVHLPKPVRVTSFS 628

Query: 181  LPRNDSFDQCNTFSTVSSSQGGEGGLGTEVVQGPWSDNVNDDENGTGFLAAYDDHGFVNG 240
            LPRNDSFDQCNTFSTVSSSQGGEGGLGTEVVQGPWSDNVNDDENGTGFLAAYDDHGFVNG
Sbjct: 627  LPRNDSFDQCNTFSTVSSSQGGEGGLGTEVVQGPWSDNVNDDENGTGFLAAYDDHGFVNG 448

Query: 241  SDFECQSHVPASDDDNSXXXXXXXXXXXXXTNDDQVQLDSFAESLLI 287
            SDFECQSHVPASDDDNS             TNDDQVQLDSFAESLLI
Sbjct: 447  SDFECQSHVPASDDDNSLEKLYEEYLQLLKTNDDQVQLDSFAESLLI 307
```

Supplemental Figure S1. Sequence analysis of other putative cacao TT2 like genes

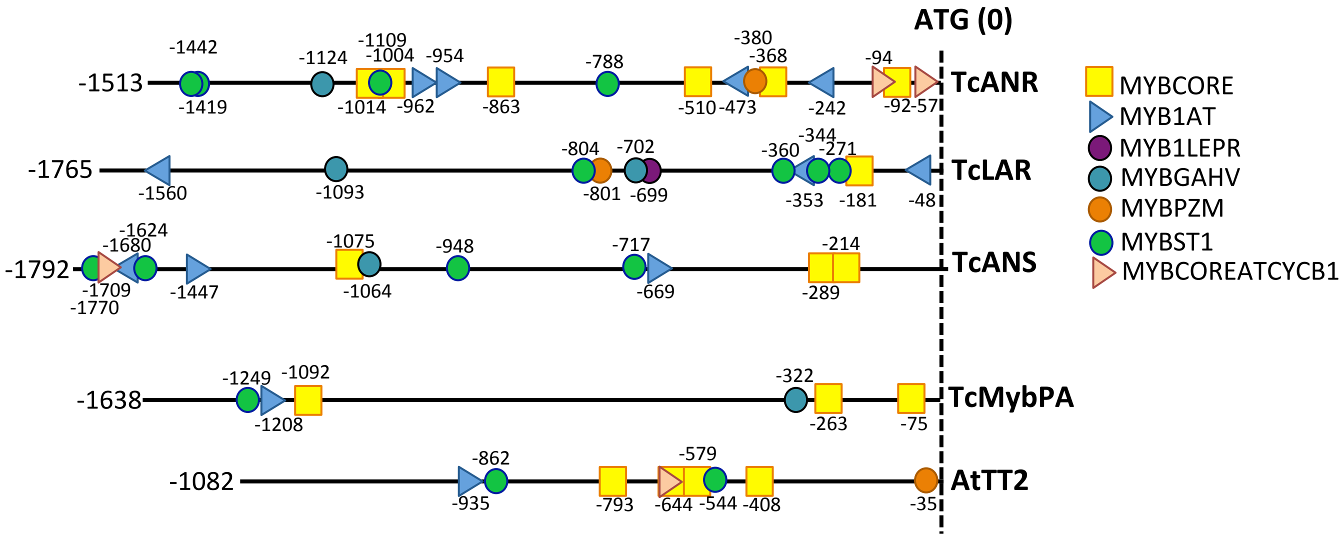

Suppmental Figure S2. MYB-binding motifs in the promoters of cacao PA synthesis genes, *TcANR, TcLAR*, *TcAN*S, transcription factor *TcMybPA* and Arabidopsis *AtTT2* detected using the PLACE database (http://www.dna.affrc.go.jp/PLACE/signalup.html).
